# Why a nuanced, pragmatic focus on yields is essential

**DOI:** 10.1098/rstb.2025.0258

**Published:** 2025-08-14

**Authors:** Andrew Balmford, Ian J. Bateman, Alison Eyres, Tom Swinfield, Thomas Ball

**Affiliations:** ^1^ Department of Zoology, University of Cambridge, Cambridge, Cambridgeshire, UK; ^2^ Land, Environment, Economics and Policy Institute (LEEP), Department of Economics, University of Exeter, Exeter, Devon, UK

**Keywords:** biodiversity, land use, farming, yield, food production

We thank Damien Beillouin and colleagues [1] for their comments. Several of the points they make align with what we tried to say in this and many previous papers—albeit perhaps not clearly enough. So to be as direct as possible:

(1) We do not advocate a continuation of business-as-usual high-yield farming. Many current practices are damaging, environmentally, socially, or both, and need to be improved or replaced by better ones.
(2) We strongly believe that farming practices need to be evaluated based on their environmental outcomes and their impacts on animal welfare and human well-being. Indeed, we have developed and applied a quantitative framework for doing so [2–4].
(3) Although increases in farm yields usually slow rates of habitat conversion (e.g. [5–8]), we agree that (as explored in [9]) maximizing the benefits of land-sparing requires additional actions to ensure effective habitat conservation, including community engagement, habitat management and legally enforced protection.
(4) We completely agree that biodiversity needs conserving in every biome. But in the context of moves by many richer nations towards increased support for farming practices that risk lowering domestic production and increasing imports from far more biodiverse parts of the world [10,11], we take the view that their efforts to enhance farmland wildlife must be accompanied by additional measures to ensure that domestic food production is maintained [12,13].

We disagree with Beillouin *et al.* on four other substantive points. First, we contend that most actions that make farmland more accommodating for biodiversity reduce yields. Beillouin and colleagues suggest otherwise, citing Jones *et al*.’s [14] analysis of 43 papers investigating biodiversity and yield outcomes of farm system diversification. But this assessment finds evidence for yields increasing with biodiversity only when the latter is measured in terms of species richness or richness-evenness. Because richness-based metrics say very little about population viability and can mask the replacement of local specialists with widespread generalists, they often generate unhelpful and in some instances contradictory results compared with more detailed analyses of how contrasting actions impact the abundance of large numbers of individually assessed species [15–18]. The Jones *et al.* analysis also looks only at impacts on species within farmed land, without reference to their status in natural habitats or indeed to the impacts of farm practices on those species (which are usually the majority) which do not live on farmed land at all: as such it is unclear how farm diversification affects biodiversity as a whole. And of course diversification describes only some of very many land-sharing practices—and even here the authors report that the most commonly recorded outcome is an increase in biodiversity but a reduction in yield. Based on this, the literature and our own and others’ analyses of the impacts of real-world practices on the abundance of approximately 2000 individually assessed species [19–27], we therefore maintain our view that practices associated with greater on-farm biodiversity are very largely associated with lower yields; and that as a result, to achieve any given level of food production they reduce the space available for natural habitats [28].

Second, we disagree that our article underestimates the problem of rebound effects, whereby increases in farm yield lower prices or raise profits and so incentivize increased production. Most local or regional studies report that rebound is indeed widespread (see above, and [29]). But Jevons effects—instances of extreme rebound where growth in total output outstrips growth in yield, resulting in the area under production increasing—appear to be rare in agriculture. Where Jevons effects are absent, yield increases are associated with reductions in natural habitat loss and so are likely to benefit biodiversity [29]. A further caution is that the magnitude of any rebound effect is likely to diminish when assessed at larger scales, because improving output efficiency in one area will, *ceteris paribus*, reduce production elsewhere. Nevertheless, reducing rebound effects in high biodiversity areas would usually be environmentally beneficial, which is why we devote space in our article and elsewhere [9,29] to exploring market and policy mechanisms that are demonstrably capable of lowering them by actively coupling yield increases with habitat protection.

A third area of disagreement concerns Beillouin and colleagues’ view that high-yield farming systems will necessarily lead to the genetic erosion of crops and related agrobiodiversity. As explained, we think it is important to consider and evaluate all agricultural systems that offer the prospect of markedly increased farm yields. Several systems—such as mixed cropping, inter-cropping, crop rotation and co-culture—offer prospects of boosting yields while increasing the diversity of varieties and breeds in use or reinstating long-established agronomic practices [30–33]. But we also note the exciting suggestion from the agrobiodiversity community that higher yield production may be key to making space, without compromising overall food production, not just for natural habitats but also areas dedicated to the maintenance even of markedly lower yielding varieties and their associated agronomic practices [34].

Finally, we believe that to identify socially just as well as environmentally beneficial outcomes, we should consider all possible high-yield farming practices. Options should only be ruled out on the basis of clear evidence that they generate unacceptable environmental or social outcomes. Beillouin *et al.* raise particular concerns about genetically modified organisms (GMOs), saying these have failed to address food security in Africa, and led to mixed outcomes in India. But both points seem poorly founded. We cannot know whether GMOs would help African food production because most African countries have not approved any GM food crop. And in India, a compelling econometric analysis confirms that growing Bt rather than conventional cotton boosts long-term yields, cuts pesticide use and increases smallholder welfare [35]—which is why almost 100% of growers continue to use Bt seeds (M Qaim, 2025, personal communication). There are, of course, important concerns about GMO adoption increasing the reliance of smallholder farmers on multinational corporations. But we see that as a reason to strongly support investments by government agencies and foundations in the development and rollout of locally appropriate GM varieties—not a justification for excluding farmers from accessing potentially groundbreaking technologies [36–38].

In sum, while acknowledging its risks, we maintain that the great bulk of evidence indicates that increasing agricultural yields is much more likely to slow biodiversity loss and mitigate climate change than approaches focused on enhancing on-farm biodiversity. We consider that on an already crowded planet where the persistence of most species and the retention of carbon-dense vegetation cannot be achieved on farmed land, the area-efficiency of food production will be a central determinant of environmental outcomes. It therefore seems sensible to evaluate the environmental and social consequences of any new or existing approaches to delivering sustained high yields, and to explore mechanisms that link support for their deployment to the effective conservation of the natural habitats on which the mitigation of the extinction and climate crises inescapably depends.

## Data Availability

This article has no additional data.

## References

[1] Beillouin D, Jones SK, Rapidel B, Estrada-Carmona N. 2025 Beyond yields: a systems approach is essential for reconciling agriculture and biodiversity. Phil Trans. R. Soc. B **1932**, 20240257. (10.1098/rstb.2025.0257) PMC12351296 40808445

[2] Balmford A *et al*. 2018 The environmental costs and benefits of high-yield farming. Nat. Sustain. **1**, 477–485. (10.1038/s41893-018-0138-5) 30450426 PMC6237269

[3] Bartlett H, Balmford A, Holmes M, Wood JN. 2023 Advancing the quantitative characterisation of farm animal welfare. Proc. R. Soc. B **290**, 20230120. (10.1098/rspb.2023.0120) PMC10031399 36946112

[4] Bartlett H *et al*. 2024 Trade-offs in the externalities of pig production are not inevitable. Nat. Food **5**, 312–322. (10.1038/s43016-024-00921-2) 38605128 PMC11045459

[5] Stevenson JR, Villoria N, Byerlee D, Kelley T, Maredia M. 2013 Green Revolution research saved an estimated 18 to 27 million hectares from being brought into agricultural production. Proc. Natl Acad. Sci. USA **110**, 8363–8368. (10.1073/pnas.1208065110) 23671086 PMC3666715

[6] Ceddia MG. 2019 The impact of income, land, and wealth inequality on agricultural expansion in Latin America. Proc. Natl Acad. Sci. USA **116**, 2527–2532. (10.1073/pnas.1814894116) 30679279 PMC6377487

[7] Villoria N. 2019 Consequences of agricultural total factor productivity growth for the sustainability of global farming: accounting for direct and indirect land use effects. Environ. Res. Lett. **14**, 125002. (10.1088/1748-9326/ab4f57)

[8] García VR, Gaspart F, Kastner T, Meyfroidt P. 2020 Agricultural intensification and land use change: assessing country-level induced intensification, land sparing and rebound effect. Environ. Res. Lett. **15**, 085007. (10.1088/1748-9326/ab8b14)

[9] Phalan B *et al*. 2016 How can higher-yield farming help to spare nature? Science **351**, 450–451. (10.1126/science.aad0055) 26823413

[10] Fuchs R, Brown C, Rounsevell M. 2020 Europe’s green deal offshores environmental damage to other nations. Nature **586**, 671–673. (10.1038/d41586-020-02991-1) 33106645

[11] Zhong H, Li Y, Ding J, Bruckner B, Feng K, Sun L, Prell C, Shan Y, Hubacek K. 2024 Global spillover effects of the European green deal and plausible mitigation options. Nat. Sustain. **7**, 1501–1511. (10.1038/s41893-024-01428-1)

[12] Bateman I, Balmford A. 2023 Current conservation policies risk accelerating biodiversity loss. Nature **618**, 671–674. (10.1038/d41586-023-01979-x) 37344644

[13] Balmford A *et al*. 2025 Time to fix the biodiversity leak. Science **387**, 720–722. (10.1126/science.adv8264) 39946469

[14] Jones SK, Sánchez AC, Beillouin D, Juventia SD, Mosnier A, Remans R, Estrada Carmona N. 2023 Achieving win-win outcomes for biodiversity and yield through diversified farming. Basic Appl. Ecol. **67**, 14–31. (10.1016/j.baae.2022.12.005)

[15] Fleishman E, Noss R, Noon B. 2006 Utility and limitations of species richness metrics for conservation planning. Ecol. Indic. **6**, 543–553. (10.1016/j.ecolind.2005.07.005)

[16] Robinson JPW, White ER, Wiwchar LD, Claar DC, Suraci JP, Baum JK. 2014 The limitations of diversity metrics in directing global marine conservation. Mar. Pol. **48**, 123–125. (10.1016/j.marpol.2014.03.012)

[17] Hillebrand H *et al*. 2018 Biodiversity change is uncoupled from species richness trends: Consequences for conservation and monitoring. J. Appl. Ecol. **55**, 169–184. (10.1111/1365-2664.12959)

[18] Fletcher R, Green R, Bladon E, Atkinson P, Phalan B, Williams D, Visconti P, Balmford A. 2025 Beyond species richness for biological conservation. Conserv. Lett.

[19] Phalan B, Onial M, Balmford A, Green RE. 2011 Reconciling food production and biodiversity conservation: land sharing and land sparing compared. Science **333**, 1289–1291. (10.1126/science.1208742) 21885781

[20] Hulme MF *et al*. 2013 Conserving the birds of Uganda’s banana-coffee arc: land sparing and land sharing compared. PLoS One **8**, e54597. (10.1371/journal.pone.0054597) 23390501 PMC3563584

[21] Gilroy JJ, Edwards FA, Medina Uribe CA, Haugaasen T, Edwards DP. 2014 Editor’s choice: surrounding habitats mediate the trade‐off between land‐sharing and land‐sparing agriculture in the tropics. J. Appl. Ecol. **51**, 1337–1346. (10.1111/1365-2664.12284)

[22] Kamp J, Urazaliev R, Balmford A, Donald PF, Green RE, Lamb AJ, Phalan B. 2015 Agricultural development and the conservation of avian biodiversity on the Eurasian steppes: a comparison of land‐sparing and land‐sharing approaches. J. Appl. Ecol. **52**, 1578–1587. (10.1111/1365-2664.12527)

[23] Dotta G, Phalan B, Silva TW, Green R, Balmford A. 2016 Assessing strategies to reconcile agriculture and bird conservation in the temperate grasslands of South America. Conserv. Biol. **30**, 618–627. (10.1111/cobi.12635) 26400720

[24] Williams DR, Alvarado F, Green RE, Manica A, Phalan B, Balmford A. 2017 Land‐use strategies to balance livestock production, biodiversity conservation and carbon storage in Yucatán, Mexico. Glob. Chang. Biol. **23**, 5260–5272. (10.1111/gcb.13791) 28614629

[25] Feniuk C, Balmford A, Green RE. 2019 Land sparing to make space for species dependent on natural habitats and high nature value farmland. Proc. R. Soc. B **286**, 20191483. (10.1098/rspb.2019.1483) PMC6732397 31455194

[26] Finch T, Gillings S, Green RE, Massimino D, Peach WJ, Balmford A. 2019 Bird conservation and the land sharing‐sparing continuum in farmland‐dominated landscapes of lowland England. Conserv. Biol. **33**, 1045–1055. (10.1111/cobi.13316) 30900280

[27] Finch T, Green RE, Massimino D, Peach WJ, Balmford A. 2020 Optimising nature conservation outcomes for a given region‐wide level of food production. J. Appl. Ecol. **57**, 985–994. (10.1111/1365-2664.13594)

[28] Green RE, Cornell SJ, Scharlemann JPW, Balmford A. 2005 Farming and the fate of wild nature. Science **307**, 550–555. (10.1126/science.1106049) 15618485

[29] Balmford A. 2021 Concentrating vs. spreading our footprint: how to meet humanity’s needs at least cost to nature. J. Zool. **315**, 79–109. (10.1111/jzo.12920)

[30] Hu L *et al*. 2016 Can the co-cultivation of rice and fish help sustain rice production? Sci. Rep. **6**, 28728. (10.1038/srep28728) 27349875 PMC4923892

[31] Tilman D. 2020 Benefits of intensive agricultural intercropping. Nat. Plants **6**, 604–605. (10.1038/s41477-020-0677-4) 32483327

[32] Brooker R *et al*. 2025 Crop mixtures: yield responses to climate and management and impacts on seed and soil chemical composition in a Scottish-based study. Plant Soil (10.1007/s11104-024-06987-y)

[33] Yang X *et al*. 2024 Diversifying crop rotation increases food production, reduces net greenhouse gas emissions and improves soil health. Nat. Commun. **15**, 198. (10.1038/s41467-023-44464-9) 38172570 PMC10764956

[34] Jago S, Borrell JS. 2024 Agrobiodiversity conservation enables sustainable and equitable land sparing. Trends Ecol. Evol. **39**, 877–880. (10.1016/j.tree.2024.08.009) 39237401

[35] Kathage J, Qaim M. 2012 Economic impacts and impact dynamics of Bt (Bacillus thuringiensis) cotton in India. Proc. Natl Acad. Sci. USA **109**, 11652–11656. (10.1073/pnas.1203647109) 22753493 PMC3406847

[36] Whitty CJM, Jones M, Tollervey A, Wheeler T. 2013 Africa and Asia need a rational debate on GM crops. Nature **497**, 31–33. (10.1038/497031a) 23636380

[37] Agre P. 2016 Laureates letter supporting precision agriculture (GMOs). See https://supportprecisionagriculture.org/nobel-laureate-gmo-letter_rjr.html.

[38] Hazell J, Jones J. 2023 Enabling genetic technologies for food security: policy briefing. London, UK: The Royal Society.

